# Introducing a sleep disorder screening and management strategy for workers with future shift work requirements: a feasibility and acceptability study

**DOI:** 10.1038/s41598-024-69479-0

**Published:** 2024-08-28

**Authors:** Brandon W. J. Brown, Robert J. Adams, Sian Wanstall, Meagan E. Crowther, Georgina Rawson, Andrew Vakulin, Tim Rayner, R. Doug McEvoy, Peter Eastwood, Amy C. Reynolds

**Affiliations:** 1 Flinders Health and Medical Research Institute (Sleep Health), Flinders University, Adelaide, Australia; 2 College of Medicine and Public Health, Flinders University, Adelaide, Australia; 3 Health Futures Institute, Murdoch University, Perth, Australia

**Keywords:** Shift work schedule, Paramedicine, Sleep initiation and maintenance disorders, Sleep apnea, Obstructive, Health services, Diagnosis, Health services

## Abstract

Sleep disorders are common, and largely undiagnosed in early-career workers. The combination of sleep disorders and shift work has implications for mental health, workplace safety, and productivity. Early identification and management of sleep disorders is likely to be beneficial to workers, employers and the community more broadly. We assessed the feasibility and acceptability of a tailored sleep disorder screening and management pathway for individuals with future shift work requirements. Paramedic students were invited to complete an online sleep health survey, which included validated sleep disorder screening questionnaires for insomnia, obstructive sleep apnea and restless legs syndrome. Participants were able to express interest in participating in a sleep monitoring and management study. Participants at risk for a sleep disorder were identified, contacted by the study physician (RJA), notified of their sleep disorder screening results and provided with information regarding management options. Feasibility of the screening and management pathways were determined by completion of the 12 week follow-up, and ability to engage with health services for diagnostic testing or treatment. Acceptability of these pathways was assessed with a semi-structured interview on completion of the study at 12 weeks. Screening was completed in thirty participants (mean age 22.5 ± 6.7, 63% female), 17 of whom were ‘at-risk’ for a sleep disorder and offered a management pathway. All participants engaged with the study physician (RJA), with 16 completing the study (94% completion rate). Three participants with excessive daytime sleepiness received feedback from the study physician (RJA) and no further care required. Of the remaining 14 participants, 11 (78%) engaged with health services after speaking with the study physician (RJA). Those who engaged with diagnostic and management services reported that a structured pathway with online screening was convenient and easy to follow. Facilitating screening and management of sleep disorders in students with future shift work requirements is both feasible and acceptable. These findings can inform the development of a preventive strategy for sleep disorders and ideally, a health services feasibility trial for future shift workers.

## Introduction

One in five (20%) young adults are living with a sleep disorder severe enough to warrant clinical investigation^1^. These sleep disorders include insomnia (the most prevalent sleep disorder in early adulthood), obstructive sleep apnea (OSA), and restless legs syndrome (RLS), and the majority are undiagnosed^1,2^. By middle age, prevalence of common sleep disorders increases to 42%^3^, and global prevalence estimates suggest that up to one billion middle aged adults are living with OSA^4,5^. First line treatment (positive airway pressure) for OSA has been shown to reduce OSA severity by as much as 86%^6^. Similarly, the preferred treatment for insomnia (cognitive behavioural therapy for insomnia [CBTi]) provides clinically significant improvements in sleep^7^. Despite having effective first line treatments for the two most prevalent sleep disorders, recent estimates suggest that many of those living with a common sleep disorder are undiagnosed, and untreated^2,8^.

Sleep disorders have considerable economic implications^9–11^. Additionally, there are health, safety and productivity consequences associated with undiagnosed and unmanaged sleep disorders, particularly in young adults. Young adults with sleep disorders report greater symptoms of anxiety and depression than those without a sleep disorder^2^. Additionally, sleep disorders result in workplace productivity loss equivalent to four working weeks a year, compared with young adults without a sleep disorder whose productivity losses equate to less than one working week per year^12^.

The combined effect of shift work *and* a sleep disorder is particularly concerning^13^. These workers are required to manage the combined impact of a sleep disorder along with varying degrees of circadian misalignment. Shift workers with a sleep disorder are more than three times more likely to report occupational burnout^14^, are twice as likely to have a motor vehicle accident^15^, are 43% more likely to make an error at work^16^, and almost three times more likely to leave their job in the first five years^17,18^. Night shift work is particularly problematic for those with a sleep disorder, with considerable impacts on mental health outcomes^2^. Yet, even when shift workers meet criteria for a sleep disorder, they are unlikely to seek help^19^.

Common sleep disorders in adults appear to affect shift workers at equal^2^ or higher^20,21^ rates to day workers, and impact almost half of the broader adult population by middle-age^3^. Consequently, there is a clear need to identify and appropriately manage sleep disorders as early as practicable. To date, no studies have considered whether early identification and management of sleep disorders *before* commencing a career with regular shift work is a feasible and acceptable option. Many careers with regular shift work require a period of education or training before commencing in professional roles. This pre-service training period provides a currently untapped opportunity to explore whether early screening and management of sleep disorders is an acceptable approach to identify, and ideally manage, sleep disorders in future shift workers.

This study aimed to determine whether offering screening and management for common sleep disorders was a feasible and acceptable strategy during the pre-employment phase for individuals entering careers which will require regular shift work.

## Methods

### Study design

This feasibility and acceptability study was conducted using a longitudinal, non-randomised design, with data collection occurring between May and November 2022. All survey questionnaires were administered using the secure Research Electronic Data Capture (REDCap) web-based platform hosted at Flinders University^22,23^. The study is reported following the CONSORT extension for pilot and feasibility studies^24^, and in accordance with guidelines for non-randomised feasibility studies^25^. Participants who completed the 12 week follow up were provided with a $100 (AUD) gift card to compensate the time required for screening, referral appointments and follow-up interviews.

### Participants

Participants were recruited from the Bachelor of Paramedicine degree at Flinders University in Adelaide, South Australia. Recruitment was undertaken via an email to all students enrolled in the degree (*n* = 390), an information flyer around campus (with a QR code and hyperlink to the survey), via the online teaching platform for the degree sequence, and during in-person delivery of a short presentation about the study from a senior researcher at commencement of coursework lectures for a two-week period. A paper copy of the information flyer was also presented to students at lectures.

Responses were reviewed by the research team in order to identify those at risk of a sleep disorder; responses and data from the ‘at risk’ participants only are reported in this manuscript. There were no exclusion criteria based on demographic or current employment information. A sleep disorder diagnosis was the only medical condition that precluded individuals from participating in the study.

### Screening questionnaires

Insomnia: The sleep condition indicator (SCI)^26^ was used to identify participants at risk for insomnia, with a cut-off of ≤ 16 and chronicity of symptoms ≥ 3 months defining an individual as being ‘at risk’.

OSA: Using the Berlin Questionnaire^27^ and Epworth Sleepiness Scale (ESS)^28^ individuals were considered to be at risk for OSA if they had two or more positive categories on the Berlin Questionnaire and an ESS score of ≥ 8.

RLS: was assessed with five questions based on the diagnostic criteria recommended by the International Restless Legs Syndrome Study Group^29^. All four symptoms had to be present and occur ≥ 5 times/month for further assessment of RLS.

Shift Work Disorder: Any individuals identified as at risk for a sleep disorder who reported working causal shift work during their studies were also screened for shift work disorder using the Shift Work Disorder Screening Questionnaire^30^. No participants met criteria for shift work disorder in this study.

Individuals with a high ESS score (≥ 10) who did not meet criteria for OSA or insomnia were also considered as ‘at risk’ and were followed up by the study physician (RJA) to rule out alternative causes of excessive daytime sleepiness beyond the common sleep disorders they were screened for (see Supplementary Material for detailed information on the assessment and management of these individuals).

### Baseline assessment

Participants were invited to the Flinders Health and Medical Research Institute (Sleep Health) for baseline anthropometrics including body weight, height, waist and neck circumference, and completed additional questionnaires (e.g. Pittsburgh Sleep Quality Index), were provided with an under-the-mattress sensor for night-to-night objective sleep monitoring (data not reported in this manuscript), and consented to participate in the study. All participants who were identified ‘at risk’ of a sleep disorder were contacted by the study physician (RJA) within a month of the baseline assessment.

### Obstructive sleep apnea diagnosis and management

Participants spoke with the study physician (RJA) and were offered a referral to a local sleep specialist for further assessment for OSA. Those who accepted a referral to the sleep specialist attended an appointment, and subsequently were referred for Level 1 laboratory-based overnight polysomnography. They were followed up by the sleep specialist for review of their results and management as indicated. Participants were offered gap-free services to reduce cost as a barrier to accessing formal diagnosis and treatment as part of this study.

### Insomnia diagnosis and management

Participants were contacted by the study physician (RJA) to confirm symptoms and chronicity, and discuss their results and recommendations for management. With consent from the participant, their general practitioner (GP) was provided with the screening results, together with information on how to refer individuals for Medicare (government) funded, first line treatment of insomnia (CBTi) with a psychologist. Additional detail about the management process for insomnia patients in this context can be found in the Supplementary Material.

### Follow up period

**Figure 1:**
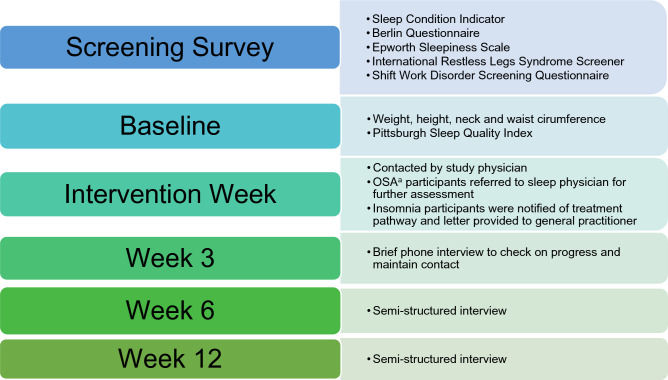
Overview of the study measurement points in participants identified as at risk of a sleep disorder. ^a^Obstructive sleep apnea.

All participants were followed up at 3, 6 and 12 weeks post call with the study physician (RJA). The follow-ups included survey questions at each time point, and a semi-structured interview at both the 6 and 12 week timepoints. A conceptual diagram of the study design is displayed﻿ in Fig. 1.

### Outcome measurement

The semi-structured interview at 12 weeks included a series of questions about the participants’ interaction with the sleep monitoring technology, action taken after being informed of having a probable sleep disorder, and their comfort with discussing their sleep in a future workplace. Participants were also asked about their general experience of the diagnosis and management pathway, and their perspective on what improvements could be made to the current services to better support prospective shift workers in the short and long term.

During the qualitative interview, researchers also explained to participants that outside of this study, there would potentially be out-of-pocket costs associated with all diagnostic and treatment modalities, even with government healthcare rebates, in order to better understand perceived barriers for future shift workers if the costs were not subsidised.

Feasibility was determined by completion of the 12 week follow up (yes or no), as well as self-reported ability of participants to access diagnostic testing (and as appropriate, treatment) for a sleep disorder during the study. Acceptability was determined with interview questions exploring their experience with health services during the trial, and their experience with the study design. A high-level summary is provided in Box 1.

Box 1 Summary of the 12 week qualitative interview questions related to feasibility and acceptability.

- You were told by our sleep physician about a potential sleep problem 12 weeks ago. Have you taken any of the recommended actions provided?
  - If Y, could you please briefly explain what action you have taken?
    - *How have you found this (relevant sleep assistance)?*
  - If N, could you please briefly explain why not?
    - *Do you feel like there is anything that makes it difficult for you to take the recommended action? Can you tell me a little more?*
- Do you feel you would be comfortable speaking about your sleep in your future workplace (ie as a paramedic)?
  - *Can you tell me why, or why not?*

Preamble: One of the goals of this study was to determine whether typical referral pathways for sleep problems are accessible for University students, and whether students are satisfied with this approach. I’m going to show you a diagram that shows a simplified example of a treatment pathway for *<  < insomnia or OSA depending on patient >  >*. In this trial, we ensured there were not out of pocket costs for sleep treatments, but we have highlighted where some of those costs might appear on the pathway in these examples.

- Can you summarise for me where you got to on this pathway?
- Can you tell me where you would like to have got to on this pathway by this point?
- Based on your own experience, which aspects of this pathway worked for you?
- Based on your own experience, what do you think are the limitations of this pathway?

### Ethics approval and consent to participate

Prior to commencement of the study, ethics approval was received from the Flinders University Human Research Ethics Committee (HREC #4813). The study was conducted in accordance with all ethics and institutional guidelines and regulations. All participants provided written, informed consent to participate in the study.

## Results

### Participants

Of the 390 potentially eligible students, 70 (18%) students commenced the screening survey, with 53 (14%) providing a complete response to all screening questions. Forty-four participants registered interest in participating in the study. Twenty-two ‘at risk’ participants according to screening were contacted and offered participation in the study, and 17 commenced. Sixteen of the 17 participants (94%) completed the 12 week follow-up. A brief voluntary exit survey link was sent to the single participant who withdrew from the study, however no further information was provided relating to their reason for withdrawal.

Demographic information is provided in Table 1. Comparison with the broader cohort including ‘low risk’ participants is provided in Supplementary Table 1. The sample comprised mostly young adults with a median (interquartile range, IQR) age of 20.5 (3.0) and a healthy body mass index (23.2 kg/m^2^ (3.0)). Participants predominantly reported working part time between 11 and 30 h per week in addition to their studies, and 6 (35%) self-reported working shift work.

**Table 1:** Characteristics of study participants identified as at risk for a sleep disorder.

	OSA^a^ (*n* = 5)	Insomnia (*n* = 9)	Excessive sleepiness (*n* = 3)	Overall (*n* = 17)	p-value^b^
Age					0.18
Median (IQR^c^)	20.0 (6)	20.0 (2)	21.0 (2)	20.0 (2)	
Sex assigned at birth					0.06
Female	1 (20.0%)	6 (66.7%)	3 (100%)	10 (58.8%)	
Male	4 (80.0%)	3 (33.3%)	0 (0%)	7 (41.2%)	
Year of study					0.54
Year one	1 (20.0%)	4 (44.4%)	2 (66.7%)	7 (41.2%)	
Year two	3 (60.0%)	3 (33.3%)	0 (0%)	6 (35.3%)	
Year three	1 (20.0%)	2 (22.2%)	1 (33.3%)	4 (23.5%)	
Weekly work (hours)					0.25
0–10 h	0 (0%)	1 (11.1%)	1 (33.3%)	2 (11.8%)	
11–20 h	3 (60.0%)	1 (11.1%)	2 (66.7%)	6 (35.3%)	
21–30 h	1 (20.0%)	5 (55.6%)	0 (0%)	6 (35.3%)	
> 30 h	1 (20.0%)	1 (11.1%)	0 (0%)	2 (11.8%)	
Unemployed	0 (0%)	1 (11.1%)	0 (0%)	1 (5.9%)	
Shift work^d^					0.22
Yes	3 (60.0%)	3 (33.3%)	0 (0%)	6 (35.3%)	
No	2 (40.0%)	6 (66.7%)	3 (100%)	11 (64.7%)	
BMI^e^					0.45
Median (IQR)	22.0 (4.1)	23.1 (2.5)	30.9 (2.1)	23.3 (6.4)	
Comorbid condition					0.13
Yes	2 (40.0%)	6 (66.7%)	0 (0%)	8 (47.1%)	
No	3 (60.0%)	3 (33.3%)	3 (100%)	9 (52.9%)	

^a^Obstructive sleep apnea, ^b^Chi-square for categorical variables and Kruskal Willis for continuous variables, ^c^Interquartile range, ^d^self reported work schedule, ^e^Body mass index (weight(kg)/height(m)^2^).

### Intervention progress

**Figure 2:**
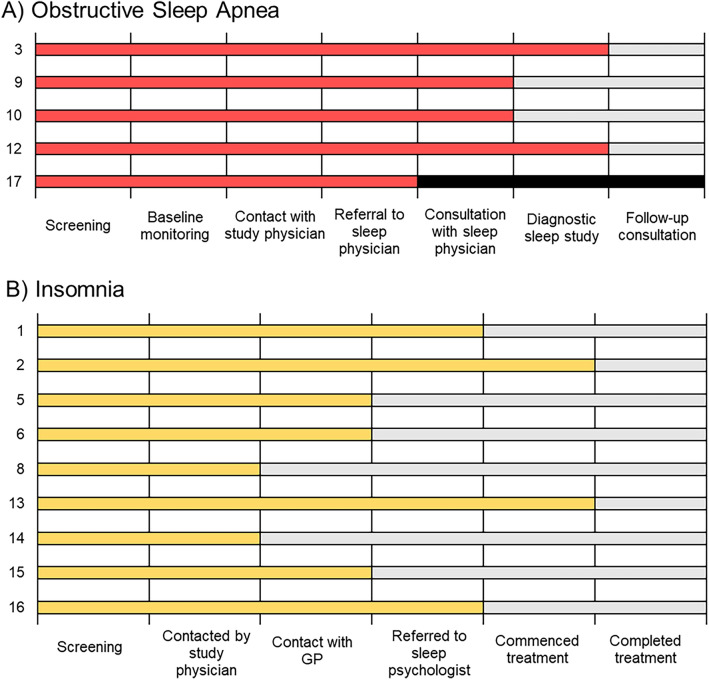
Participant progress for OSA and insomnia management during the 12 week follow up period. (**A**) red colour denotes OSA participant progress at the 12 week follow up, and (**B**) yellow colour denotes insomnia participant progress at the 12 week follow up. Participant ID is on the y axis. ^a^General Practitioner, *Participant 17 withdrew prior to completing the study, black colour denotes progress not possible beyond withdrawal.

Individual participant progress over the 12 weeks is outlined in Fig. 2. Participants were randomly numbered 1–17 to protect identity. Progress with health services varied by participant, and by presenting sleep disorder risk. All participants (*n* = 17) engaged in a conversation about being ‘at risk’ for a sleep disorder with the study physician (RJA), and referral pathways were discussed.

The three participants with excessive sleepiness were contacted by the study physician (RJA) and received a telehealth appointment. Insufficient sleep opportunity and resultant sleep restriction were identified as the most likely cause of excessive sleepiness. These participants were provided with information regarding healthy sleep habits, and the study physician (RJA) determined no further action was required with health services for these participants.

The remaining results relate to participants at risk for insomnia and OSA (*n* = 14). Overall, 86% (12/14) of these participants had engaged with health services related to their sleep after speaking to the study physician (RJA). For OSA, this was a minimum of attending a consultation with a sleep physician, and for insomnia this was making contact with a GP which is the required first step towards achieving subsidised sleep services in Australia.

Two participants in the insomnia group had no further health services contact after the initial discussion with the study physician (RJA, 2/14, 14%). Three made contact with their GP but had not yet taken further action at the 12 week follow up. Four participants (4/14, 29%) had received a referral to see a sleep psychologist, and two of these participants (2/14, 14%) were referred and commenced treatment (CBTi). One of these participants came close to completion of treatment (CBTi) within the 12 week follow-up.

Four of the five participants in the OSA group (4/5, 80%) had attended an initial consultation with a sleep physician for OSA to discuss diagnosis pathways with a sleep study, and two had subsequently attended an overnight diagnostic sleep study (2/5, 40%). The remaining participant withdrew after speaking with the study physician (RJA).

## Screening feasibility

Many participants spoke directly about the ease of online screening, and feeling that this approach addressed a barrier to seeking help. The process was broadly described as ‘easy’ or ‘simple’.

*[H]aving someone reach out to us was definitely easier. I think if it was going to be that we had to initiate the process, I think I probably wouldn’t have, so I’m glad that it was so simple.* (Participant 13).

*If I wasn’t in the study I would have – well, probably the first bit recognising there was an issue and even going to somebody. But because I was in this study and the screening process was done for me it made it all a lot easier and then it all just flowed on from there.* (Participant 12).

*I don’t know if it's just our cohort or all paramedic students, but we’re pretty bad at like help seeking kind of behaviour. So I think the way that this study reached out to us was good. Like we got an email that said, “If you would like to participate, click this link,” or something. So that it meant that we didn’t even have to be help seeking, we kind of just had to click a link.* (Participant 13).

One participant pointed towards a lack of initiative by students to seek help for health conditions, and described barriers that may influence behaviour. When talking about help seeking more broadly, accessing help was perceived as being associated with stigma within the workplace, possibly impacting future job opportunities.

*With the trauma side of things or like feeling anxious or like PTSD [post-traumatic stress disorder] kind of related stuff, it makes you think that you're less likely to get a job opportunity if you reach out on a [employing organisation] side of things. There’s definitely a fear that that will get like a red cross next to your name.* (Participant 13).

## Feasibility of referral and management for sleep disorders

### Obstructive sleep apnea

Feasibility of the referral pathway for OSA for connecting participants to appropriate health services was evidenced by engagement with sleep physicians for specialist care. Of the five participants identified as ‘at risk’ for OSA, four (80%) attended a face-to-face appointment with a sleep physician for review. Two of the four subsequently attended an overnight sleep study within the 12 week study window.

The fifth participant was referred to a sleep physician for review, but then withdrew from the study. Further management of OSA was not captured within the 12 week study window for any of the participants, even with two completing overnight diagnostic studies.

Qualitative feedback from the participants indicated that the referral process for OSA ***diagnosis*** was both feasible and acceptable. However, engaging with the overnight diagnostic sleep study was perceived as more challenging for some participants. Specifically, for the two participants who did not have diagnostic sleep studies during the study window, making time to stay overnight at the sleep laboratory was the main barrier.

*Since then [appointment with sleep physician] with everything going on in my life I haven’t been able to organise coming in here to do an actual study either. *(Participant 9).

*I think I was saying after exams when I can afford to stay a night.* (Participant 10).

These two participants still wanted to pursue the diagnostic study, but achieving this during the 12 week study period was not as feasible given their individual circumstances.

Content and relevance of the in-person sleep physician consultation was questioned by one participant, who stated:

*I was expecting a lot more from it. It was just kind of like [the physician] looked in my throat, listened to my chest and then said, “Yeah, we’ll get in touch about a sleep study here” and that was about it.* (Participant 9).

Of the other participants reviewed by the sleep specialist, two reported that the consultation provided insight into treatment options and risk factors for OSA, whilst the final participants did not comment directly on the physician appointment.

### Insomnia

Of the participants who were identified as ‘at risk’ for insomnia (*n* = 9), seven (78%) made contact with their GP in line with the recommended pathway to access Australian Government funded (Medicare), psychological services on a mental health care plan under the *Better Access* scheme^31^. Of these seven, four received a referral for CBTi treatment with a psychologist (44% of those ‘at risk’ for insomnia), a further two (22%) had made contact with a psychologist to commence first line treatment (CBTi) and one participant had an appointment with their GP within the 12 week follow up period but had not progressed further.

### Referrals for insomnia

For the two participants who did not engage with a GP to discuss referral to a sleep psychologist, making time to go and see their GP was identified as the key barrier. Students indicated they had limited time during standard business hours which made booking an appointment difficult due the operating hours of their GP. Travel time was also described as an additional barrier, especially in the context of work and university commitments during business hours.

*Just timing for me personally, like with uni and working and like kind of living on opposite sides of the city to uni. It’s really hard, like taking in travel time. I’m never really available within working hours. So it’s always hard for me to go to the doctor.* (Participant 14).

Seven of the nine participants at risk for insomnia did speak to their GP about a referral for CBTi, with mixed outcomes. All participants at risk for insomnia reported having a regular GP, although with varying frequency of usual interaction. While all nominated GPs received a template letter from the study physician (RJA) describing the results and options for referral and management, not all participants received a referral to a sleep psychologist.

Some participants reported having no trouble with the referral process.

*So I had an appointment already but it was easy just to be able to bring this up as well, so that she could kind of just add that on. And she said it was super easy because you get like 20 sessions under mental with care plan [increased session availability due to COVID19], which is great. So there was no like barriers to being able to get an appointment or anything.* (Participant 13).

*“Hey, can I go and see this person?” And she was like, “Yeah, sounds good” and just like, “That’s just the mental health care plan” and I was like, “Awesome”.* (Participant 16).

This was not the case for all participants, with the interaction with the GP identified as a barrier to accessing CBTi.

*…I had a sleep psychologist referral as well and he was like, “Oh, we’re out of time” and I’ve never had a doctor tell me that we’re out of time truly in a very, very long time.* (Participant 1).

*…it was good from this end [study referral]. Like the email that was like all the steps that, like it was very easy to follow. I think if I was with a different GP [it] would’ve been heaps easier and heaps quicker.* (Participant 13).

One barrier which can impact the feasibility of this referral and management pathway was specific to participants with pre-existing mental health care plans. Existing mental health care plans affected the ease of access to additional psychology services specifically for sleep under this scheme. One participant discussed prioritising their current treatment plan over seeking help for their sleep.

*The thing is, I was using up my mental health plan for my own stuff before that, and so at the risk of kind of using that up for sleep data, it wasn’t particularly attractive to me. *(Participant 6).

However, another participant was able to have their current care plan amended to access treatment for their sleep.

*I already had one, I’m pretty sure, so it was just that it had to be adapted a little bit or he did up another one. *(Participant 1).

### Accessing treatment for insomnia

Two participants did receive a referral to a psychologist, but did not commence treatment within the 12 week window. One had misplaced the referral and was unable to obtain another during the study timeframe, whilst the other reported having insufficient time to contact psychological services for an appointment at the 12 week follow up.

Two participants commenced CBTi within the 12 week follow up. Both identified that the ability to access treatment for insomnia through telehealth made access easier. One participant did report a preference for in-person consultations, although did not feel as though their treatment was of lower quality when delivered via telehealth.

*It was really convenient actually to just sort of use like the telehealth website and stuff like that and it made it so much easier* (Participant 2)

Treatment efficacy was not assessed for this study, however, both participants who were receiving treatment independently reported feeling more confident in their ability to manage their sleep within the 12 week follow up.

*I think what I’m doing now, like with the treatment with the psychologist, having strategies that would make – I don’t know if I do, but make me feel like I have more control over my sleep, which will take the pressure off because I know that I’ve got things I can put in place to give me the best shot possible of having a good sleep.* (Participant 13).

## Overall acceptability of a screening, diagnosis and treatment pathway facilitated during tertiary education

All but one participant felt that the screening, diagnosis and treatment pathway presented was acceptable. One concern raised was the recommendation to access treatment for insomnia on a mental health care plan, with a participant feeling a referral for insomnia may interfere with their ability to access treatment for other mental health concerns. The remaining participants either reported no concerns about the process, or stated they found it acceptable. Online screening was described as an easy process, and participants felt the ability to talk about the results over the phone with the study physician was helpful and preferrable to an in-person appointment.

*I like the whole process of screening, get some results, refer on over the phone* (Participant 10).

External factors were most commonly cited as the limiting factor in seeking help once the risk of a sleep disorder was identified. For the two participants who received referrals but did not access treatment, both thought that this was achievable in the given study duration, with their personal circumstance being the only barrier.

*This is a completely achievable treatment pathway and I don’t think you could simplify it anymore, like all the tools are given to you. I don’t know if people maybe didn’t see a bulk-billed GP or something if it would be maybe like a financial thing or something but I feel like it’s pretty accessible.* (Participant 1).

Participants felt that one of the appealing aspects of this study was that costs normally associated with accessing diagnostic testing (sleep physician consultation and diagnostic sleep study) and treatment (CBTi) did not carry an out-of-pocket expense. These findings are highlighted by one participant who stated that they were previously recommended to have a sleep study and were interested in pursuing this, but cost was a limiting factor.

*I wanted to do a sleep study, but it was going to cost me a lot of money.* (Participant 17).

There was a mixed response when discussing the likelihood of accessing treatment for a sleep disorder, such as purchasing a Continuous Positive Airway Pressure (CPAP) device for OSA, if they were to incur out of pocket costs. One participant thought that this was not something most young adults would consider a necessary expense.

*…CPAP machines are a couple grand [two thousand dollars] sometimes, so it’s like the 26 year old uni student’s going to drop two grand [two thousand dollars] on a mask they have to wear at night, probably not. Because let’s say you kind of get to that point where you’re just like, “Oh, I’m healthy, I’m fine, I'm invincible” type situation.* (Participant 9).

The cost associated with healthcare was described as a justifiable expense for some participants, particularly when they felt their sleep problem had persisted for a while.

*Depending on how much that gap [co-payment] was, I don’t think it would’ve stopped me from going through with it because it’s something that’s been going on for years.* (Participant 2).

*I wouldn’t want to spend two grand [two thousand dollars] on absolutely nothing but if it helped, there would be no hesitation. *(Participant 3).

This sentiment was not shared by all participants, with some feeling cost was a barrier to help seeking. Participants identified that healthcare may not be a priority for students more broadly, making them less likely to engage if there are associated costs.

*Personally, I always get put off by costs. That’s with anything. My GP has gone private as well so I’m probably going to find a new GP* (Participant 5).

*Cost would probably definitely be a barrier and if we already knew that it was going to be expensive, I think people just wouldn't even initiate it.* (Participant 13).

## Discussion

Screening for sleep disorders and providing a cost subsidised treatment pathway is both feasible and acceptable in a cohort of tertiary education students with future shift work requirements. Students were able to engage with screening, sleep physician review, and in a small number of cases initiate treatment, within a 12-week window. Broadly, students felt that the ease of access with online screening, coupled with rapid review, meant they received care which may have been more challenging to access if they were required to self-refer and find time to attend appointments. However, barriers to engagement with health services were apparent at both the individual and health system levels. This made accessing treatment more challenging for some participants. Together, these findings provide support for screening, referral and management for sleep disorders *before* completion of education or training for careers which have future shift work requirements. The identified barriers and enablers are particularly important to address in future studies, given 20% of young Australian adults are living with a clinical sleep disorder, and > 80% are undiagnosed^1,2^.

This study was intentionally designed to support referral and treatment which aligned with the current Australian healthcare system for sleep problems, including screening questionnaires (OSA) and use of mental health care plans (insomnia) which facilitate access to government subsidised services^32,33^. The referral pathway was consistent between OSA patients, with scheduling difficulties cited as the main reason for not participating in an overnight (diagnostic) sleep study to confirm a diagnosis. On the other hand, the recommended treatment pathway for insomnia required consultation with a GP to attain a referral to a psychologist for frontline treatment (CBTi) with government subsidies. Participants reported challenges with obtaining the correct referrals, as well as miscommunication and confusion around the processes required to access government subsidised care, as some of the barriers to accessing treatment during the study. Although the referral process is not without its challenges for GPs who are often time poor, completion of a mental health care plan and referral to a psychologist is common practice for Australian GPs. This challenge may be specific to insomnia, and reflect broader sentiments about complexity with managing insomnia referrals in the primary care setting^33^.

Although all participants were navigating the same healthcare system, there were notable differences in accessing treatment for insomnia within our modest pilot sample. At present, the government initiative provides patients with up to 10 subsidised sessions (annually) with a mental health practitioner following referral from a GP. This pathway is commonly utilised for access to psychological support for depression and anxiety, however, a recent study by Haycock et al.^33^ identified that inconsistencies remain around awareness that insomnia is an eligible condition under the scheme.

An additional important finding from this study was the challenge of accessing treatment for insomnia by individuals who were already utilising psychological services on a mental health care plan for other conditions. This barrier is of particular importance given that not all psychologists are experienced with management of sleep disorders^34^. As a result, while a current treating psychologist may be suited for management of broader mental health presentations, there may be additional need for a psychologist with competency in the management of insomnia. Yet, the current system may struggle to facilitate this option with an affordable service, especially considering managing insomnia was perceived in our participants as a lower priority relative to other mental health concerns. This is an interesting dilemma more broadly, especially given that effectively treating sleep disorders like insomnia has been shown to reduce symptoms of anxiety and depression^35^.

Together, these findings suggest a need for multiple intervention targets, including education and awareness about the bidirectional nature of sleep and mental health, and advocacy for referral pathways which allow subsidised sleep management without the barriers associated with existing treatment subsidy schemes. Online sleep disorder symptom screening was successfully implemented in this study, in line with a large study of healthcare workers^36^. Providing access to online screening and clear information about possible diagnosis and treatment pathways for future shift workers is a low cost intervention which could assist with improving awareness on sleep disorders, and provide a pathway to access help. This aligns with recommendations from shift workers with sleep disorders, who felt that having easily accessible information was an important step in the pathway to effective treatment^13^.

The translation from screening to diagnosis and management is more challenging. Our findings suggest that implementing pre-shift work sleep disorder screening and management strategies are likely to be negatively impacted if relying solely on government-supported and rebated health services. Consideration should be given to lower cost and more rapidly accessible services for future shift workers, such as diagnostic sleep testing at home and telehealth consultations, which have become common practice in Australia.

Time constraints were commonly reported as a barrier to help seeking by participants, irrespective of the presenting sleep disorder. This is not uncommon, particularly in young adults where time demands impact the priority they give to their sleep more broadly^37^. While students can be time poor due to the combination of study, work and social commitments, motivation may also have been a contributing factor for those who did not seek help and ultimately, receive treatment. Rates of help seeking for sleep are often low more broadly, especially in young adults. A large postal survey conducted by Bartlett et al.^38^ found that only 6% of individuals under the age of 25 years old with insomnia had seen their GP regarding their sleep. Further, in a representative community cohort of young, working Australian adults, less than 20% of those who met criteria for a common sleep disorder reported they had received a sleep disorder diagnosis^2^. Of note in our study, only 18% of the potentially eligible students participated in the sleep health survey on their sleep and even fewer completed the survey (14%). We were not able to determine in this study what drives low rates of engagement for help seeking more broadly, and future studies should better explore the contributing factors to low engagement in young adults in the population.

Although there is limited population-level insight into rates of help seeking in existing literature, participants in our study expressed difficulty in making appointments with their GP as the consultation hours often overlapped with their work and study commitments. For participants at risk of OSA who did not have a diagnostic sleep study during the study, external commitments including social obligations and work were reported as the main barriers. As the added requirement to spend a night in the sleep laboratory for diagnostic polysomnography was logistically inconvenient for some participants, at home testing was mentioned as a possible solution to address this barrier. Another consideration is at which stage of change participants may have been during the study. According to the transtheoretical model, an individual will progress through a series of stages before successfully modifying a behaviour (in this instance, effective engagement with health services for sleep)^39^. While we did not directly assess this, it is plausible that participants in this study who did not progress to making appointments or engaging in sleep treatment may still fall within the ‘precontemplation’, ‘contemplation’ or ‘preparation’ stages. The impact of poor sleep on an individual’s stage of change, subsequent behaviour^40^ and motivation for change^41^ have been previously explored, and provide insight into the complexities of help seeking for a sleep problem. In future studies, tailoring interventions to the individual’s current stage could be beneficial for improving health services engagement specifically for sleep disorders.

Healthcare costs were predominantly subsidised for this study, with the only costs likely to be co-payments for GP appointments to receive referrals. To determine whether this had an impact on help seeking, participants were asked during interviews what influence cost has on their healthcare decision making. Cost was an important factor when considering access to healthcare, with participants often referring to the impact of low income associated with being a university student. Interestingly, one participant reported that they were likely going to seek out a new GP as their current provider no longer offers a completely bulk-billed service (e.g. no co-payment for consultation). However, a recent national report identified that only 35% of Australian GP clinics offer bulk billing services, with rates as low as 25% in South Australia^42^.

Accessing treatment through private health cover may be another option in Australia for a portion of private psychology fees. However, it is unclear how many participants in this study had private health cover, or would be willing to seek help through private healthcare providers for their sleep, particularly when there is still likely to be a co-payment. There can be a significant cost associated with management (CPAP, oral appliances) for OSA, and in young adults this may be prohibitively expensive without both financial support, and further education on the long-term benefit of treatment. Future interventions will need to consider the economic barriers over a longer period of time to better understand and inform recommendations specifically for sleep disorder management in young adults.

As the intent of this study was to assess the feasibility and acceptability of a tailored screening and referral process for sleep disorder management, longer-term treatment related outcomes were not reported. This study reported participant experience through qualitative interviews, providing a unique insight into screening and help seeking processes experienced by students through a sleep clinic co-located at their university campus. Whilst this is a strength of the study, the experience of our participants who had access to an on-site sleep service will likely differ from other cohorts where sleep services may not be as easily accessible. Additional considerations include the use of a non-randomised approach which could result in recruitment bias. However, this intentional recruitment strategy was needed to ensure that participants in the study met criteria for sleep disorders, as the focus was on accessing health services, rather than treatment effectiveness. Consideration of both OSA and insomnia is a strength of the study, as we were able to highlight that pathways through the healthcare system are markedly different according to diagnosis.

## Conclusion

Sleep disorder screening and management pathways are feasible and acceptable in the tertiary education setting for students with future shift work requirements. Implementation of a consistent, transparent and accessible referral process, which minimises cost to ensure this is not a prohibitive barrier to accessing diagnosis and treatment, would appear to be the most acceptable approach. Given that there are economic, organisational and individual level consequences associated with unmanaged sleep problems, this early intervention approach is likely to be beneficial to organisations, as well as their new employees. These pilot findings suggest a need for future randomised controlled trials to identify whether early intervention for sleep disorders in students with future shift work requirements translate into effective management. Such approaches may offer an innovative and cost-effective strategy to assist with the transition to shift work, and improve health and safety outcomes of workers with regular shift work requirements in their future professions.

### Supplementary Information

Supplementary Information.

## Data Availability

The data collected and/or analyzed during the current study are not publicly available in accordance with ethical approvals required, but may be available on reasonable request and subject to consultation with the relevant human ethics committee. Contact corresponding author Brandon Brown (brandon.brown@flinders.edu.au) for any request to access data from this study.
