# Supplementary material for: Introducing a sleep disorder screening and management strategy for workers with future shift work requirements: a feasibility and acceptability study
Source: Sci Rep. 2024 Aug 28;14:19964. doi: 10.1038/s41598-024-69479-0 (PMC11358459; doi:10.1038/s41598-024-69479-0)
Supplement: Supplementary file 1 — Supplementary Information. [file 41598_2024_69479_MOESM1_ESM.docx]

**Supplementary Materials**

Introducing a sleep disorder screening and management strategy for workers with future shift work requirements: A feasibility and acceptability study

Brown, B.W.J*^1^; Adams, R.J^1^; Wanstall, S^1^; Crowther, M.E^1^; Rawson, G^1^; Vakulin, A^1^; Rayner, T^2^; McEvoy, R.D^1^, Eastwood, P^3^; Reynolds, A.C.^1^.

Affiliations

^1^Flinders Health and Medical Research Institute (Sleep Health), Flinders University, Adelaide, Australia; ^2^College of Medicine and Public Health, Flinders University, Adelaide Australia; ^3^Health Futures Institute, Murdoch University, Perth, Australia

*Corresponding author: Brandon Brown, brandon.brown@flinders.edu.au

**Methodology**

Obstructive Sleep Apnea Diagnosis

In Australia, the Medicare Benefits Schedule currently has two pathways for an individual to access a diagnostic study for obstructive sleep apnea (OSA). General practitioners are able to refer individuals to a sleep specialist for further assessment or directly to a diagnostic sleep study if they meet the referral pathway rules. To meet this criteria, individuals must have a positive OSA screening questionnaire (Berlin Questionnaire [BQ], OSA50 or STOP-BANG) and an Epworth Sleepiness Scale (ESS) score ≥8 ^1^.

Berlin Questionnaire

The BQ assesses risk for OSA with 11 questions across three categories including, snoring (category 1), daytime sleepiness (category 2) and, obesity (body mass index [BMI]), and hypertension (category 3). Category 1 includes four questions (1 point each) assessing snoring including presence, frequency per week (≥3 nights per week), severity (≥ louder than talking) and disruption to others, and one question (2 points) regarding witnessed apnoeas (≥3 nights per week) ^2^. Category 2 assesses daytime symptoms across three questions (1 point each) including fatigue upon wakening (≥3 days per week), daytime fatigue (≥3 days per week) and history of falling asleep whilst driving. Category 1 and 2 are scored individually and are positive if there is a score of 2 or more in each respective category. Category 3 is positive if the respondent indicates they have hypertension or if their BMI is ≥30kg/m2. Individuals who are positive in any two categories are considered high risk for OSA.

Epworth Sleepiness Scale

The ESS provides an estimation of daytime sleepiness through questions assessing the likelihood of nodding off across eight different scenarios. Each question is scored on a scale of 0-3 with higher scores indicating greater levels of sleepiness ^3^. An ESS score of ≥10 was used as an indicator of clinically significant daytime sleepiness.

Chronic Insomnia

Sleep Conditions Indicator

The Sleep Conditions Indicator (SCI) is an eight item questionnaire developed based on the insomnia disorder diagnostic criteria in the Diagnostic and Statistical Manual of Mental Disorder, Fifth Edition ^4^. Across the eight items, four elements of sleep are assessed including, sleep continuity (falling asleep and staying asleep), satisfaction with sleep (quality and concern about sleep), frequency and chronicity and, daytime consequences. The scoring of the SCI ranges between 0 to 32 with higher scores representing better sleep. An SCI score of ≤16 indicates high risk for insomnia.

Restless Legs Syndrome

Restless Legs Syndrome (RLS) was assessed with five questions (four regarding symptoms, one assessing chronicity) based on the diagnostic criteria recommended by the International Restless Legs Syndrome Study Group. The questions assessed urge to move your legs when sitting or lying down, presence of discomfort during the urge to move your legs, whether the discomfort is relieved by walking or moving your legs, the level of associated distress, and whether the urge causes disruption to sleep. Participants were identified at risk of RLS if they reported the presence of all four symptoms ≥5 times per month.

Shift Work Disorder

Participants who identified working shift work during their studies and were ‘at risk’ of a sleep disorder were assessed for shift work disorder (SWD) using a validated screening questionnaire ^5^. The SWD screening questionnaire assesses an individual’s risk across four questions relating to the impact of non-standard shifts on sleep, wellbeing and, likelihood of falling asleep at work, or while driving after at least two days off from work. Each question is assigned a score from 1 to 4 and is multiplied by a classification function coefficient and the constant added, to determine either high or low risk of SWD.

Excessive Sleepiness

During review of the initial screening results, a subset of participants were identified as having excessive daytime sleepiness (ESS ≥10) without meeting criteria for OSA or insomnia. The decision to include these individuals despite not meeting diagnostic criteria for the included sleep disorders was based on the clinical judgement of the study physician (RJA), to ensure that less prevalent sleep disorders were not overlooked in potentially at-risk individuals. These participants were individually contacted by the study physician for further assessment of other sleep conditions that were not screened for (e.g. idiopathic hypersomnolence, narcolepsy, parasomnias) or the possibility of OSA which was not detected by the screening questionnaire.

Insomnia management (additional detail)

In Australia, under the Better Access scheme, insomnia is an eligible condition for access to subsidised treatment from a psychologist ^6^. In order to avoid costs associated with treatment which might impact the University students referred, GPs were provided with referral details for the insomnia treatment program at the Adelaide Institute for Sleep Health, and participants who chose this pathway had access to treatment with CBTi with no associated co-payment. Participants and their GPs were under no obligation to follow these recommendations, and participants were able to engage with an alternative provider or treatment pathway if this was their preference.

Table S1 – Demographic characteristics of all participants included in the study

|  | **OSA^a^ (*n*=5)** | **Insomnia (*n*=9)** | **Excessive Sleepiness (*n*=3)** | **‘Low risk’ Sleepers (*n*=13)** | **Overall (*n*=30)** | **p-value^b^** |
| --- | --- | --- | --- | --- | --- | --- |
| **Age** |  |  |  |  |  | 0.11 |
| Median (IQR^c^) | 20.0 (6) | 20.0 (2) | 21.0 (2) | 21.0 (3) | 21.0 (3) |  |
| **Sex** |  |  |  |  |  | 0.11 |
| Female | 1 (20.0%) | 6 (66.7%) | 3 (100%) | 9 (69.2%) | 19 (63.3%) |  |
| Male | 4 (80.0%) | 3 (33.3%) | 0 (0%) | 4 (30.8%) | 11 (36.7%) |  |
| **Year of study** | |  |  |  |  | 0.66 |
| Year one | 1 (20.0%) | 4 (44.4%) | 2 (66.7%) | 4 (30.8%) | 11 (36.7%) |  |
| Year two | 3 (60.0%) | 3 (33.3%) | 0 (0%) | 4 (30.8%) | 10 (33.3%) |  |
| Year three | 1 (20.0%) | 2 (22.2%) | 1 (33.3%) | 5 (38.5%) | 9 (30.0%) |  |
| **Weekly work (hours)** | |  |  |  |  | 0.27 |
| 0-10 | 0 (0%) | 1 (11.1%) | 1 (33.3%) | 2 (15.4%) | 4 (13.3%) |  |
| 11-20 | 3 (60.0%) | 1 (11.1%) | 2 (66.7%) | 8 (61.5%) | 14 (46.7%) |  |
| 21-30 | 1 (20.0%) | 5 (55.6%) | 0 (0%) | 3 (23.1%) | 9 (30.0%) |  |
| >30 | 1 (20.0%) | 1 (11.1%) | 0 (0%) | 0 (0%) | 2 (6.7%) |  |
| Unemployed | 0 (0%) | 1 (11.1%) | 0 (0%) | 0 (0%) | 1 (3.3%) |  |
| **Shift work^d^** |  |  |  |  |  | 0.36 |
| Yes | 3 (60.0%) | 3 (33.3%) | 0 (0%) | 6 (46.2%) | 12 (40.0%) |  |
| No | 2 (40.0%) | 6 (66.7%) | 3 (100%) | 7 (53.8%) | 18 (60.0%) |  |
| **Neck circumference (cm)** | |  |  |  |  | 0.11 |
| Median (IQR) | 35.0 (3.5) | 33.0 (4.8) | 36.5 (1.5) | 33.0 (3.5) | 34.0 (4.4) |  |
| **BMI^e^** |  |  |  |  |  | 0.47 |
| Median (IQR) | 22.0 (4.1) | 23.1 (2.5) | 30.9 (2.1) | 23.1 (2.8) | 23.2 (3.0) |  |
| **Comorbid condition** | |  |  |  |  | 0.05 |
| Yes | 2 (40.0%) | 6 (66.7%) | 0 (0%) | 2 (15.4%) | 10 (33.3%) |  |
| No | 3 (60.0%) | 3 (33.3%) | 3 (100%) | 11 (84.6%) | 20 (66.7%) |  |
|  |  |  |  |  |  |  |

Note, ^a^Obstructive sleep apnea, ^b^Chi-square for categorical variables and Kruskal Willis for continuous variables, ^c^Interquartile range ^d^self reported work schedule, ^e^Body mass Index (weight(kg)/height(m)^2^)

References

1. Hamilton, G. S. & Chai-Coetzer, C. L. Update on the assessment and investigation of adult obstructive sleep apnoea. *Aust J Gen Pract* **48**, 176–181 (2019).

2. Netzer, N. C., Stoohs, R. A., Netzer, C. M., Clark, K. & Strohl, K. P. Using the Berlin Questionnaire to identify patients at risk for the sleep apnea syndrome. *Ann. Intern. Med.* **131**, 485–491 (1999).

3. Johns, M. W. A new method for measuring daytime sleepiness: the Epworth sleepiness scale. *Sleep* **14**, 540–545 (1991).

4. Espie, C. A. *et al.* The Sleep Condition Indicator: a clinical screening tool to evaluate insomnia disorder. *BMJ Open* **4**, e004183 (2014).

5. Barger, L. K. *et al.* Validation of a questionnaire to screen for shift work disorder. *Sleep* **35**, 1693–1703 (2012).

6. Haycock, J. *et al.* Primary care management of chronic insomnia: a qualitative analysis of the attitudes and experiences of Australian general practitioners. *BMC Fam. Pract.* **22**, 158 (2021).
